# Oasis 2: improved online analysis of small RNA-seq data

**DOI:** 10.1186/s12859-018-2047-z

**Published:** 2018-02-14

**Authors:** Raza-Ur Rahman, Abhivyakti Gautam, Jörn Bethune, Abdul Sattar, Maksims Fiosins, Daniel Sumner Magruder, Vincenzo Capece, Orr Shomroni, Stefan Bonn

**Affiliations:** 1 Laboratory of Computational Systems Biology, German Center for Neurodegenerative Diseases, Göttingen, Germany; 2 Institute of Medical Systems Biology, Center for Molecular Neurobiology, University Clinic Hamburg-Eppendorf, Hamburg, Germany; 3 German Center for Neurodegenerative Diseases, Tübingen, Germany

## Abstract

**Background:**

Small RNA molecules play important roles in many biological processes and their dysregulation or dysfunction can cause disease. The current method of choice for genome-wide sRNA expression profiling is deep sequencing.

**Results:**

Here we present Oasis 2, which is a new main release of the Oasis web application for the detection, differential expression, and classification of small RNAs in deep sequencing data. Compared to its predecessor Oasis, Oasis 2 features a novel and speed-optimized sRNA detection module that supports the identification of small RNAs in any organism with higher accuracy. Next to the improved detection of small RNAs in a target organism, the software now also recognizes potential cross-species miRNAs and viral and bacterial sRNAs in infected samples. In addition, novel miRNAs can now be queried and visualized interactively, providing essential information for over 700 high-quality miRNA predictions across 14 organisms. Robust biomarker signatures can now be obtained using the novel enhanced classification module.

**Conclusions:**

Oasis 2 enables biologists and medical researchers to rapidly analyze and query small RNA deep sequencing data with improved precision, recall, and speed, in an interactive and user-friendly environment.

**Availability and Implementation:**

Oasis 2 is implemented in Java, J2EE, mysql, Python, R, PHP and JavaScript. It is freely available at https://oasis.dzne.de

**Electronic supplementary material:**

The online version of this article (10.1186/s12859-018-2047-z) contains supplementary material, which is available to authorized users.

## Background

Small RNAs (sRNAs) are a class of short, non-coding RNAs with important biological functions in nearly all aspects of organismal development in health and disease. Especially in diagnostic and therapeutic research sRNAs, such as miRNAs and piRNAs, received recent attention [18]. The current method of choice for the quantification of the genome-wide sRNA expression landscape is deep sequencing (sRNA-seq).

To date several local as well as server-based sRNA-seq analysis workflows are available that differ in their analysis portfolio, performance, and user-friendliness. Analysis workflows that need to be installed by the end-user comprise, for example, sRNA workbench [1] for the quantification and identification of differentially expressed sRNAs and CAP-miRSeq [16] for the quantification of known and novel miRNAs including variant calling and subsequent differential expression analysis. While workflows that are installed on a local machine offer greater data security and may provide greater flexibility, they require installation, availability of servers, software and hardware maintenance as well as regular updates.

Recent additions to sRNA analysis web applications include omiRas [11], supporting quantification, differential expression and interactive network visualization; mirTools 2.0 [20] that allows for differential expression and gene ontology analysis of detected sRNAs; MAGI, an all-in-one workflow with detailed interactive web reports [8]; Chimira that allows for the detection of miRNA edits and modifications [17]; sRNAtoolbox [15] performs expression profiling of sRNA-seq data, differential expression as well as target gene prediction and visualization of analysis results; and Oasis [2], which supports the detection and annotation of known and novel sRNAs, multivariate differential expression analysis, biomarker detection, and job automation via an advanced programming interface (API). Here we present Oasis 2, an improved major release of the Oasis web application with many new and enhanced features for Biologists and Bioinformaticians (Table 1).

**Table 1:** sRNA-seq web application comparison

Feature	Oasis 2	Oasis	omiRas	mirTools 2.0	MAGI	Chimira	sRNAtoolbox
FASTQ compression	✓	✓			✓	✓	
miRNA prediction	✓	✓	✓	✓	✓		✓
miRNA modifications and edits						✓	✓
Novel miRNA database	✓						
Infection and cross-species analysis	✓						✓
Non-model organism	✓					✓	
Differential expression	✓	✓	✓	✓	✓	✓	✓
Multivariate differential expression	✓	✓					✓
Classification	✓	✓					
Novel miRNA target prediction	✓	✓		✓	✓		✓
Pathway/GO analysis	✓	✓	✓	✓	✓		✓
Batch job submission (API)	✓	✓					
Genome browser							✓

Of note, this comparison does not include all available sRNA analysis web applications. It only considers the most recent web applications that we deemed most competitive and we do not compare to standalone software solutions that have to be locally installed

At the heart of Oasis 2 lies the new sRNA detection workflow that is faster and identifies more sRNAs with higher precision. In addition, Oasis 2 now supports sRNA-seq analyses for any organism, detects potential cross-species miRNAs, and reports viral and bacterial infections in samples with high precision and recall. Oasis 2 predicts and stores novel miRNAs in Oasis-DB and allows users to search and extract information for over 700 predicted high-quality miRNAs across 14 organisms. Oasis 2 classification module is improved with the use of balanced sampling and feature pruning methods that enables robust biomarker detection. Like its predecessor Oasis, Oasis 2’s differential expression module supports multiple group comparisons (e.g. control vs. treatment 1 vs. treatment 2) and differential expression using co-variates such as age, gender, and medication. The differential expression and classification modules report various quality metrics including known and predicted targets of miRNAs in a downloadable, interactive web report. This web report allows for the subsequent functional enrichment analysis of miRNAs using GeneMania (interactome and GO analysis) [21], g:Profiler (GO, pathway-Kegg, Reactome) [13], STRING (protein-protein interaction network) [4], STITCH (chemical-protein interaction network) [9], and DAVID (enrichment analysis based on many biological databases) [6]. Oasis 2 is also at the heart of the sRNA Expression Atlas (SEA, https://sea.dzne.de), a web application for the interactive querying, visualization, and analysis for over 2000 published sRNA samples. Lastly Oasis 2 features many new analysis and visualization options such as support for adapter trimmed data, options to trim additional barcodes, and interactive plots for sRNA detection and classification output. It has no restrictions on the size or number of samples and has no limits on the analyses per user.

## Implementation

The following paragraphs will describe the technical details of Oasis 2’s novel sRNA detection, database, and classification modules. Additional information can be found in the supplementary material.

### sRNA detection

**Fig. 1:**
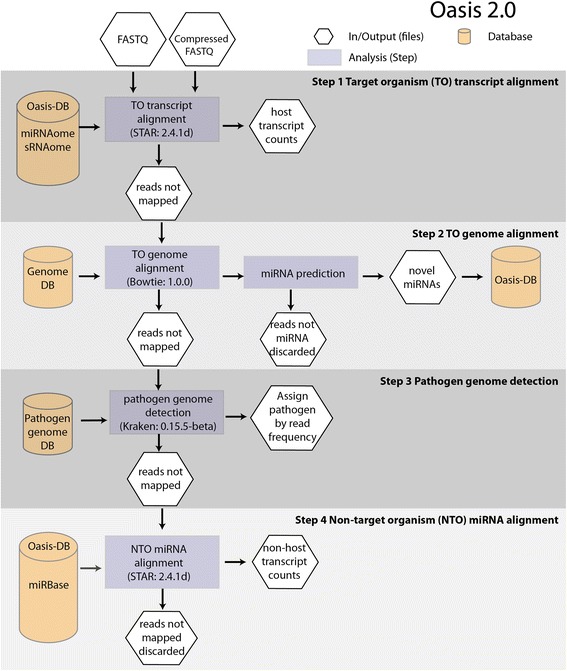
Detection of sRNAs in Oasis 2: The web application allows for the upload of raw or compressed FASTQ files to Oasis 2’s sRNA detection module. After pre-processing (adapter/barcode trimming and length filtering), reads are first aligned to target organism (TO) transcripts that are stored in Oasis-DB (Step 1), including known miRNAs, piRNAs, snoRNAs, snRNAs, rRNAs, and high-stringency predicted miRNAs and their families. Unmapped reads of Step1 are subsequently aligned to the TO’s genome (Step 2) to predict and subsequently store novel miRNAs in Oasis-DB. Unmapped reads from step 2 are mapped to bacterial, archaeal, and viral genomes using Kraken (Step 3) to detect potential pathogenic infections or contaminations. Finally, reads that could not be aligned in steps 1–3 are aligned to all non-target organism (NTO) miRNAs in miRBase (Step 4) to detect potentially orthologous or cross-species miRNAs. In case the user’s data does not correspond to one of the 14 supplied organisms, Oasis 2 aligns the reads only to NTO miRNAs (Step 4), supporting the detection of miRNA expression in any organism

**Fig. 2:**
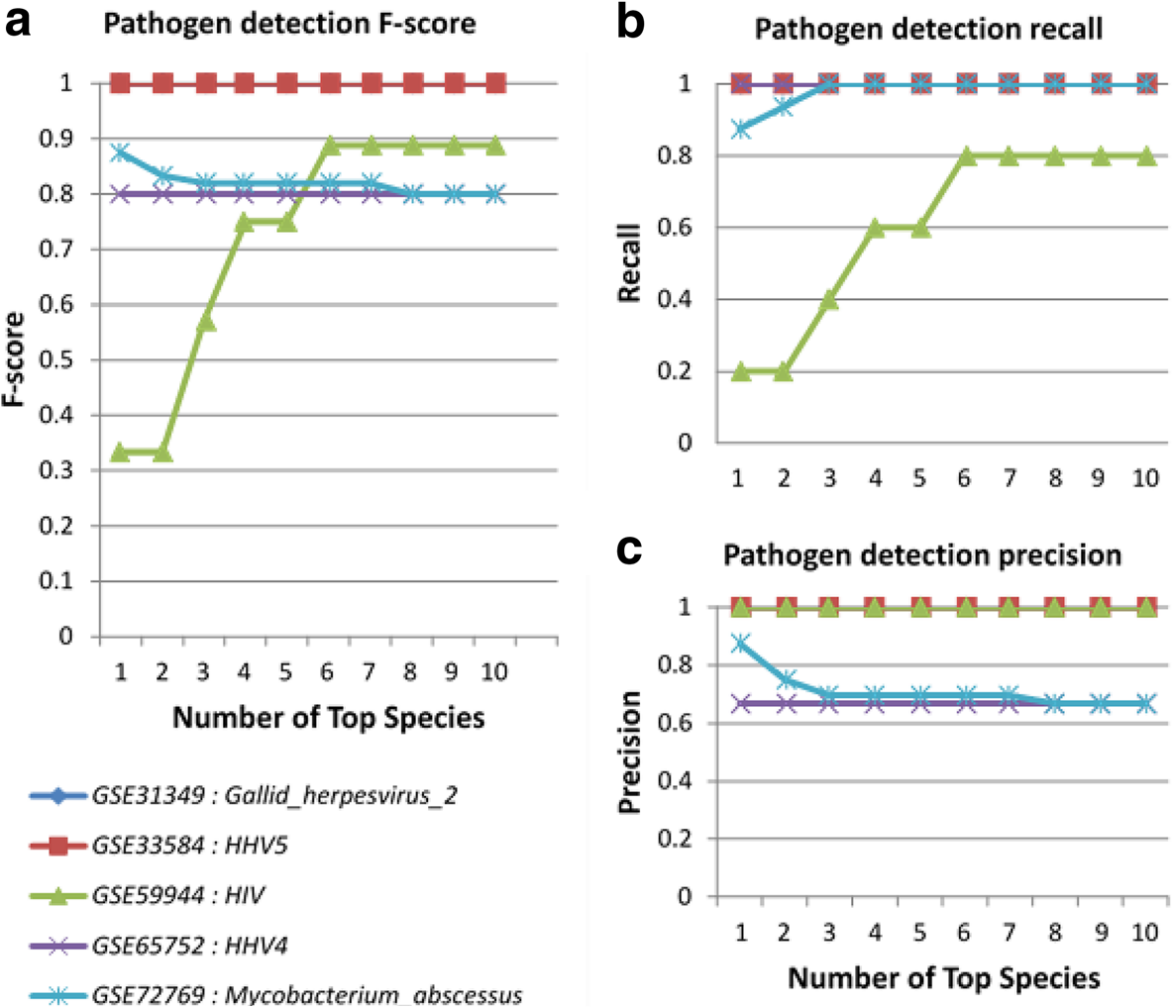
Pathogen detection performance: To assess the performance of ‘pathogen detection module’, sRNA datasets with defined viral or bacterial infections were analyzed and the F-score (**a**), recall (**b**), and precision (**c**) of the pathogen predictions were measured for the top 10 reported organisms. Overall, the prediction of bacterial (*M. abscessus*) and viral (*HIV, HHV4, HHV5, Gallid_herpesvirus_2*) infections resulted in high F-scores, recall, and precision, especially when the top 5 predicted pathogen species are reported. In consequence, Oasis 2 currently reports the top five predicted pathogen species based on their read counts

One of the key differences between Oasis 2 and its predecessor is the fully revised detection of known and novel sRNAs. The new detection workflow increases the alignment speed, is more accurate, and supports the analysis of any model and non-model organism (Fig. 1, Additional file 1). While Oasis detected sRNAs using a single genome alignment step, Oasis 2 is based upon a four-tiered alignment strategy. Users can upload (un)-compressed data that originates from one of the 14 different organisms provided in Oasis 2 and the data will be aligned to the (i) target organism’s (TO) transcripts, (ii) TO’s genome, (iii) pathogen genomes, and (iv) non-target organism’s (NTO) miRNA transcripts in succession (Fig. 1). In the TO Transcript alignment (step 1), reads are aligned to TO transcripts in Oasis-DB, a database that contains transcript information of miRNAs and other sRNA species (snRNA, snoRNA, rRNA and piRNAs) from miRBase, piRNAbank, Ensembl, predicted novel miRNAs, and sRNA families. In this step reads of length 15–19 nucleotides are aligned with no mismatches whereas reads of length 20–32 nucleotides are mapped allowing for 1 mismatch (Step 2 in Fig. 1). In the TO Genome alignment (step 2), reads that do not align to TO transcripts are subsequently aligned to the reference genome allowing for 1 mismatch and no more than five potential genomic target regions to predict novel, high-quality miRNAs (Additional file 1 section 1.2 ‘Alignment and counting’). Predicted novel miRNAs are then added to Oasis-DB as described in section 2.2 ‘Detection and storage of novel miRNAs’. In the Pathogen Genome detection (step 3), reads that could not be aligned to the TO transcriptome or TO genome are used to identify pathogenic sRNA signatures from bacteria and viruses, supplying information on potentially infected samples (Fig.2 & Additional file 1). To this end, we indexed Oasis Pathogen-Genome-DB that consists of 4336 viral and 2784 bacterial/archaeal genomes with Kraken [19] using a k-mer length of 18. In the Non-TO miRNA alignment (step 4), reads that could not be aligned to TO transcripts, the TO genome or pathogen genomes are aligned without any mismatches to all NTO transcripts of miRBase to detect potential orthologous or cross-species miRNAs. In cases where the data does not belong to one of the 14 supported genomes available in Oasis 2, reads can be aligned to all known and novel predicted miRNAs and miRNA families stored in Oasis-DB (Additional file 1).

In addition to the new alignment strategy, the sRNA detection module also supports data with already trimmed adapters. It also has an option for barcode removal, which is required for the analysis of libraries generated with e.g. the NEXTflex kit. In the case of barcode removal, Oasis 2 first discards the 3′ adapter sequence (in case the adapter is not already trimmed), and then removes an additional N (user defined, default is 0) bases from the adapter-clipped reads.

### Detection and storage of novel miRNAs

Another major improvement of Oasis 2 is the ability to query and visualize detailed information for over 700 high-quality predicted miRNAs across 14 organisms (Fig. 1, Additional file 1: Figure S1). Oasis-DB comprises information on all MiRDeep2 [5] predicted miRNAs that pass stringent selection criteria during the sRNA detection step of Oasis 2 (2.1 & Additional file 1), including the miRNA ID, organism, chromosomal location, precursor and mature sequences, structure, read counts, prediction scores, and detailed information on the software and its versions used to predict the miRNA. To assure that Oasis-DB contains only high-quality miRNA entries, novel predicted miRNAs have to pass the three criteria. The log-odds score assigned to the hairpin by miRDeep2 (miRDeep2-score) should be greater than 10, the predicted miRNA hairpin should not have sequence similarity to reference tRNAs or rRNAs, and the estimated randfold *p*-value of the excised potential miRNA hairpin should be equal to or lower than 0.05.

Novel predicted miRNAs are added to Oasis-DB using the standard nomenclature (Additional file 1 section 1.4 ‘Oasis-DB miRNA insertion and naming’).

In addition to novel miRNAs, Oasis-DB also stores information on all other sRNAs and sRNA families (Additonal file 1). To provide access to Oasis-DB we created a novel web frontend, the Oasis 2 ‘Search’ module, which allows users to query miRNAs by mature/precursor ID or sequence, and the organism they come from. Information on high-confidence novel miRNAs is also shared with SEA, a web application that provides expression information of known and novel miRNAs for over 2000 samples (https://sea.dzne.de).

### Classification and differential expression

To allow for enhanced sRNA-based biomarker detection several profound changes to the Oasis 2 classification module were made, resulting in more robust biomarker detection with increased accuracy (Additional file 1: Figure S2 , Additional file 1 section ‘Oasis 2 classification module’). To increase the performance of the Random Forest-based (RF) classification module we first implemented balanced sampling (Additional file 1), making sure RF predictions would not be biased in the case of uneven class distribution. Since RFs can perform poorly on data that contains few informative and many non-informative features, the classification module was augmented with a feature pruning routine (Additional file 1), reporting prediction performance for the full and best RF models. In addition to providing information on model accuracy using the out-of-bag (OOB) error, Oasis 2 now also provides model performance information based on cross-validation. All classification results can be explored in interactive web reports, allowing for a detailed quality and performance analysis of the predicted biomarkers.

Moreover, we have improved the quality of output plots in the DE module and updated the DESeq2 version for the analysis of differential sRNA expression. Further details about DE module can be found in Additional file 1 section 1.5 ‘Oasis 2 differential expression module’ and Additional file 1: Table S3.

### Technologies and compatibility

Oasis 2 is implemented in Java, J2EE, mysql, Python, R, PHP and JavaScript. For the usage JavaScript should be enabled in the browser. Oasis 2 functionality was tested on all major browsers (Table 2). It has no restrictions on the size or number of samples and has no limits on the analyses per user. Potential user-specific problems can arise when i) an institution or university has upload limits, ii) proxy settings that would interrupt or prohibit long uploads, or iii) JavaScript is disabled or blocked. Oasis 2 is freely available at (https://oasis.dzne.de).

**Table 2:** Oasis 2 browser compatibility

Browser	Version
Chrome	61.0.3163.100, 62.0.3202.62
Mozilla Firefox	55.0.3, 56.0 (64-bit), 57.0 (64-bit)
Chromium	62.0.3202.75
Safari	11.0.1
Internet explorer	11

Browsers that are used to test Oasis 2 functionalities

## Results

We compared the set of analysis options and the analysis speed of Oasis 2 to six state-of-the-art sRNA analysis web applications, including Oasis, omiRas, mirTools 2.0, MAGI, Chimira and sRNAtoolbox, and found that it compares favorably in the number of analysis options (Table 1) and the analysis speed (Table 3). When tested on four publically available datasets, Oasis 2 detected 19 out of 27 (70%) differentially expressed (DE) genes that were previously validated (true positives) and did not detect 4/4 (100%) miRNAs that showed a significant DE in deep sequencing but could not be validated with qPCR (false positives), highlighting both the sensitivity and specificity of Oasis 2. Finally, we compared the performance of the novel classification module to the one implemented in Oasis, showing that prediction accuracy as well as robustness are increased.

**Table 3:** Runtime comparison of different sRNA-seq web applications

Demo Dataset	Oasis 2 (total) ^1^	Oasis (total)^1^	MAGI (total)	Chimira (total)	omiRas	mirTools^7^ 2.0	sRNAtoolbox
AD (287 GB)^4^	8 h31m50s	12h29m12s	NA^2^	NA^4^	NA^5^	NA	NA
Psoriasis (48 GB)	1h35m17s	5h49m4s	48h^3^	3h3m12s	NA^6^	NA	NA
Renal Cancer (9 GB)	31m43s	1h8m41s	8h^3^	47m11s	9h31m	NA	NA

^1^Run time estimate includes the data compression and decompression, the sRNA Detection, DE Analysis, and Classification. ^2^ We could not get MAGI to upload all AD files. Most probably it has a problem with the quality or format of one of the files. ^3^ These values were obtained from the MAGI website. ^4^ Chimira does not support the analysis of more than 25 files at a time, which prohibited us from getting runtime estimates for the AD dataset. ^5^ omiRas did not finish uploading files, which prohibited us from getting runtime estimates for the AD dataset. ^6^ omiRas http uploading error. ^7^ We cannot compare the runtime of mirTools 2.0 as maximum file size to upload is limited to 30 Mb. The sRNAtoolbox web application has been non-functional since 30/05/2017, which prohibited any runtime comparison (http://bioinfo2.ugr.es:8080/srnatoolbox/quick-start/)

### Detection and differential expression of sRNAs

To estimate if the novel sRNA detection workflow of Oasis 2 identifies and quantifies sRNAs correctly we analyzed four published datasets containing validated sRNA changes using Oasis 2 with default settings. Of note, none of the above-mentioned publications looked into the DE of other small RNA classes (snRNA, snoRNA and rRNA and piRNAs), so the analyses were restricted to miRNAs.

#### Alzheimer disease data

We started by analyzing an Alzheimer disease (AD) sRNA dataset that consists of 48 Alzheimer and 22 control samples [10] using Oasis 2 and default settings. The original publication uses a Wilcoxon-Mann-Whitney test detecting 125 known DE miRNAs. Oasis 2 detected 103 DE miRNAs using an adjusted *p*-value < 0.1, of which 62(60%) overlapped with the original analysis. The overlap of 60% seems reasonable, given the different statistical approaches and miRBase versions used for the detection and DE analysis of the miRNAs. In the original publication 8/10 known miRNAs were validated to be differentially expressed in the same direction, whereas two miRNAs (hsa-miR-1285-5p and hsa-miR-26a-5p) were not validated in the same direction (instead of upregulation they showed downregulation in qPCR). Interestingly these two miRNAs were not detected to be differentially expressed by Oasis 2. On the other hand Oasis 2 was able to detect 3/3 upregulated miRNAs (hsa-let-7d-3p, hsa-miR-5010-3p and hsa-miR-151a-3p), 3/5 downregulated miRNAs (hsa-miR-532-5p, hsa-miR-26b-5p and hsa-let-7f-5p), and it did not detect two downregulated miRNAs (hsa-miR-103a-3p, hsa-miR-107). In summary, Oasis 2 was able to detect 6/8 (75%) validated differentially expressed known miRNAs and not detecting 2/2 false positives from the original study. Unfortunately, two novel miRNAs validated in the original study are not added to miRBase yet, therefore we were not able to compare to them.

#### Psoriasis data

Oasis 2’s performance was next assessed using a set of 10 Psoriasis and 10 control samples [7]. The original publication uses a hypergeometric test to assess differential expression (Pearson’s chi-square test) that is followed by a Bonferroni multiple-testing correction.

In accordance with the analyses performed in the original publication, we only considered non-redundant pre-miRNAs. Oasis 2 found 195 DE miRNAs (166 non-redundant known pre-miRNAs) (adjusted *p*-value < 0.1) whereas the original publication contains only 98 DE miRNAs (70 non-redundant known pre-miRNAs). Of the 70 DE pre-miRNAs in the original study, 51 (72.85%) could also be found in the list of Oasis 2 DE miRNAs (Table 4). In addition, 5/8 (62.5%) experimentally validated DE miRNAs (miR-21, miR-31,,, miR-944, miR-135band miR-675) were detected by Oasis 2, not identifying validated miRNAs miR-124, miR-431 and miR-219-2-3p that show high expression variation in the original publication. Furthermore, Oasis 2 identified 2/3 (67%) predicted novel DE miRNAs (hsa-miR-203b and hsa-miR-3613) while missing hsa-miR-4490 (miRBase v21). In addition, Oasis 2 did not detect the false positive miR-431* (1/1, 100%) that was predicted to be DE in the original Psoriasis study [7] but could not be validated by qPCR. In summary, Oasis 2 was able to detect 7/11 (64%) validated differentially expressed known and novel miRNAs and did not detect the only available false positive miRNA from the original study.

**Table 4:** Overlap of differentially expressed sRNAs using three datasets

	Statistic^1^	Overlap^2^	Validated overlap^3^	FP overlap^4^
AD	Wilcoxon-Mann-Whitney	60%	75%(6/8)^5^	0% (0/2)
Psoriasis	Pearson’s chi-squared	73%	64% (7/11)	0% (0/1)
Renal Cancer	edgeR [14]	76%	80% (4/5)	NA
Schizophrenia	DESeq2 (Dejian et al., 2015)	41%	67%(2/3)	0% (0/1)

^1^Oasis 2 uses a negative binomial distribution as basis for its statistical evaluation of the differential expression. A very similar approach is taken by the edgeR package that has been used in the Renal Cancer study. The Psoriasis data was analyzed using a Pearson’s chi-squared test and the AD dataset was analyzed using the non-parametric Wilcoxon-Mann-Whitney test. Schizophrenia dataset used the same approach like Oasis 2. ^2^Overlap of differentially expressed miRNAs comparing Oasis 2’s results to published data. The percentage is calculated in reference to the shorter DE list. ^3^Overlap of differentially expressed miRNAs that have been validated independently in addition to the sRNA-seq experiment. ^4^False positive (FP) differentially expressed miRNAs detected by Oasis 2. ^5^Only known validated DE miRNAs are considered

**Fig. 3:**
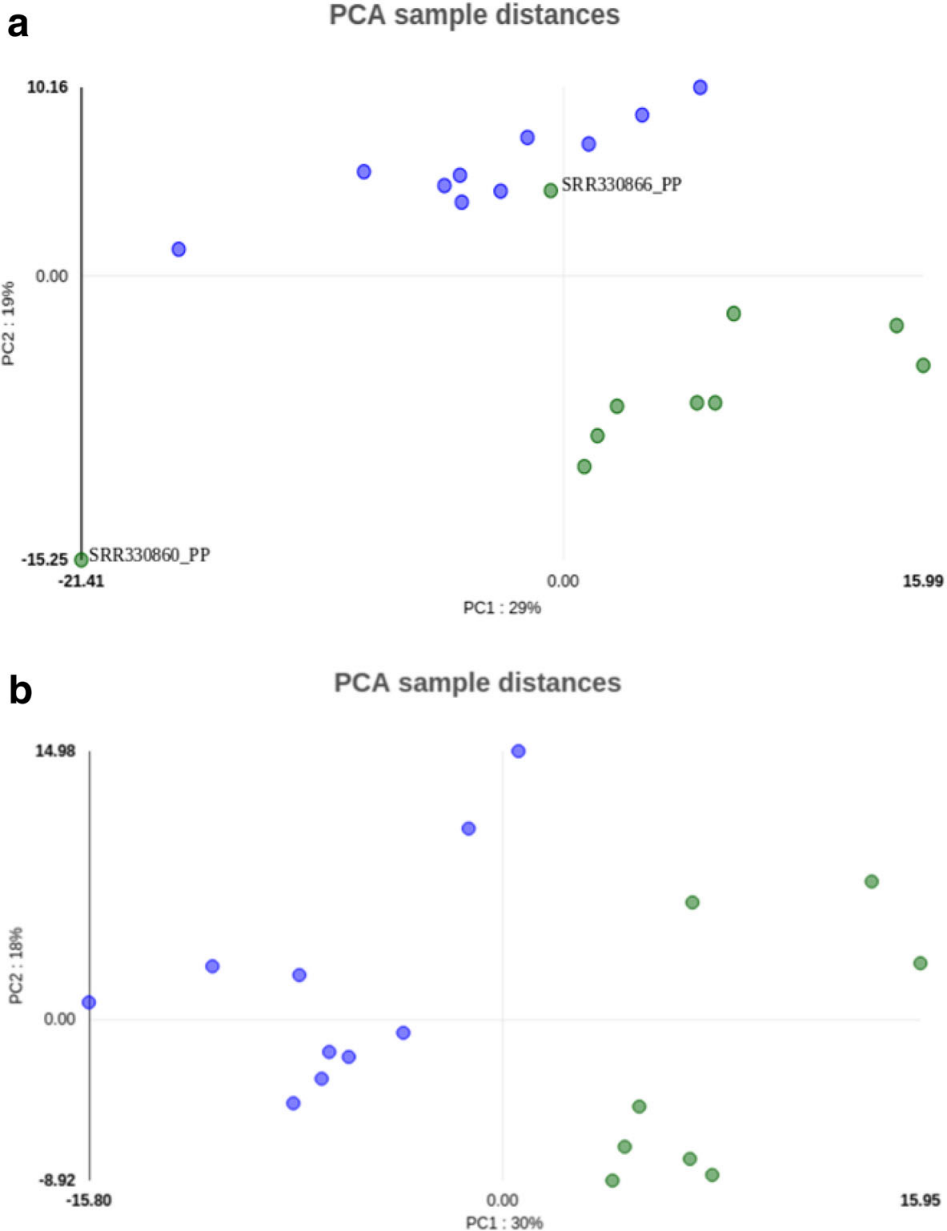
Oasis 2′ (QC) outlier detection: To assess the QC of Oasis 2 and its biological relevance, sRNA Psoriasis data (demo dataset) was analyzed. PCA sample distances of psoriasis (green) and control (blue) is shown. (**a**) PCA of psoriasis and control samples showing a potentially mis-annotated (SRR330866_PP) and an outlier sample (SRR330860_PP). (**b**) PCA of psoriasis and control samples without misclassified/outlier samples. Removal of these two samples increased the number of significantly (adjusted *p*-value < 0.1) DE miRNAs from 195 to 256 cases and increased the AUC from 0.9 to 1 in the classification module, providing strong evidence for the utility of Oasis 2’ QC plots

Of note, Oasis 2’ PCA analysis highlights a potentially mis-annotated Psoriasis sample and another outlier sample (Fig. 3A). Removal of these two samples (Fig. 3B) increased the number of significantly (adjusted *p*-value < 0.1) DE miRNAs from 195 to 256 cases. We would like to emphasize that this data was already analyzed in two publications and to our knowledge this is the first time that these ‘problematic’ samples were detected, providing strong evidence for the utility of Oasis 2’ QC plots.

#### Renal cancer data

In this work 11 renal cancer and 11 remission samples [12] were analyzed. This is longitudinal data from 11 patients and as such paired but we were unable to extract the pairing information from the GEO database annotations. Therefore the data was analyzed with Oasis 2 in un-paired mode and compared to the published, paired analysis with edgeR [14]. Despite of these technical issues the two analyses showed high overlap. Oasis 2 found 150 DE miRNAs (adjusted *p*-value < 0.1) whereas the original publication lists only 70 DE miRNAs. Of these 70 DE miRNAs 53 (76%) could also be found in the significant Oasis 2 miRNAs (Table 4). Of note, with the exception of miR-122 all the validated miRNAs from the original work were detected using Oasis 2 (miR-21-5p, miR-210-3p, miR-199, miR-532-3p).

#### Schizophrenia and schizoaffective disorder data

In this experiment induced pluripotent stem cells were used to study neuropsychiatric disorders associated with 22q11.2 microdeletions [3]. Controls and patients with 22q11.2 microdeletions diagnosed with a psychotic disorder were compared (9 controls and 7 patients). Oasis 2 found 34 DE miRNAs (adjusted p-value < 0.1) whereas the original publication identified 45 DE miRNAs. Of these 45 DE miRNAs 14 (41%) were also detected as differentially expressed by Oasis 2 (Table 4). In the original publication four miRNAs were validated by qPCR, two significantly up-regulated (miR-23a-5p and miR-146b-3p), one significantly down-regulated (miR-185-5p), and a miRNA that showed no difference in expression (miR-767-5p). Oasis 2 was able to confirm 2/3 (67%) validated differentially expressed miRNAs (miR-23a-5p and miR-185-5p) and did not confirm 1/1 (100%) false positive miRNAs miR-767-5p.

Overall, Oasis 2 detected 19/27 (70%) independently validated DE miRNAs in the published datasets despite of the different statistical approaches and miRBase versions used (Table 4). Detailed analysis results are accessible in Oasis 2’s ‘Demo Data’ webpage. Our results provide strong evidence that Oasis 2 provides biologically meaningful results to the end user.

### Pathogen detection and sample classification

To assess the performance of the pathogen detection we analyzed 5 datasets with known viral or bacterial infections (Additional file 1: Table S6). We calculated the precision, recall, and F-score for the detection of the particular pathogen strain in the dataset while considering only the top ranking, first two, three, and up to the first ten reported species (Fig. 2). Species were ordered based on the number of read counts. In general, the viral or bacterial species and strains were detected with high precision and recall, reaching F-scores of ~ 0.8 when the top five viral and bacterial species were considered. In consequence, Oasis 2 currently reports the top five bacterial, archaeal, and viral species found, allowing for the detection of potential infective agents or the discovery of experimental sample contaminations.

To benchmark the improved classification routine, we compared the performance of the old Oasis classification module (unbalanced sampling with all variables) to the new Oasis 2 classification module using balanced sampling and feature optimization using three demo datasets (see Detection and Differential Expression of sRNAs and Additional file 1: Figure S2). From a theoretical perspective, balanced sampling should increase prediction accuracy only in the case of class imbalances. In consequence, the novel classification module enhances the AUC for the imbalanced AD (22 controls, 48 patients) demo dataset by 2% (old AUC 0.95, new AUC 0.97), while it marginally changes classification performance for the balanced Psoriasis (10 control and 10 Psoriasis samples) (old AUC 0.90, new AUC 0.91) and Renal carcinoma (11 control and 11 cancer samples) (new and old AUC 1.00) data. Feature pruning should be crucial when a dataset contains a lot of uninformative features and very few informative features. To this end we have taken an unpublished dataset (6 controls, 6 treatments) that contains at least one feature that perfectly separates the two classes but otherwise contains mostly uninformative features. Whereas the old classification module reaches an AUC of 0 on this dataset, the new module reaches an AUC of 0.833.

Moreover, we also compared the accuracy of the new Oasis 2 classification module on the AD dataset to the published accuracy in the original manuscript [10]. Unfortunately, we were unable to obtain the primary output of the SVM and could not follow the post-processing steps of the machine learning results as performed in the original publication (e.g. removal of miRNAs that also occur in other diseases). In brief, the original publication provides a biomarker signature of 12 miRNAs (10 annotated and two novel) that reaches an average accuracy of 80%. The Oasis 2 classification reaches an accuracy of ~ 87% (AUC of 0.97) using 320 features (no preprocessing for other diseases) and has an out-of-bag error of ~ 10%. Two miRNAs in the original paper list (has-miR-151a-3p, hsa-let-7f-5p) were also found in the top 10 features (miRNAs) obtained with Oasis 2 classification.

The classification analysis of the three demo datasets (see 3.1) yielded stable and robust biomarker predictions that further corroborated the quality of the enhanced classification module.

### Runtime estimates

We next estimated the runtime of Oasis 2 using the above-mentioned AD, Psoriasis, and Renal cancer datasets and compared the results to runtime estimates for omiRas, mirTools 2.0, MAGI, Chimira and sRNAtoolbox, five recently developed web applications for the analysis of sRNA-seq data (Table 3, Additional file 1: Table S7). Performances of the sRNA Detection, DE Analysis, and Classification modules were measured on the Oasis 2 server. For benchmarking the Oasis 2 runtime we compared it to the runtime estimates of the above-mentioned web applications by submitting the AD, Psoriasis, and Renal Cancer datasets to the respective services (Table 3). Of note, runtime estimates for MAGI were taken from the MAGI webpage, which we assume constitutes a ‘best case scenario’ in favor of MAGI (low server analysis load). In addition, we could not compare to mirTools 2.0 as the maximum upload file size is limited to 30 Mb. Furthermore, the sRNAtoolbox web application was also not accessible during the period of testing and writing this manuscript.

Overall, Oasis 2 is significantly faster than MAGI, Chimira, and omiRas. For the smallest dataset (Renal Cancer) Oasis 2 was ~ 1.5 times faster than Chimira, ~ 15 times faster than MAGI, and ~ 18 times faster than omiRas. While the runtime differences between Oasis 2 and Chimira were rather small when only few samples were analyzed, Oasis 2 was ~ 2 times faster than Chimira, ~ 30 times faster than MAGI for the 48 Gb Psoriasis dataset. Unfortunately, we were unable to estimate the runtime of omiRas for the Renal Cancer dataset since it did not finish file upload. Oasis 2 analyzed the largest dataset (AD, 287 Gb) in 8 h31m50s while none of the other tools mentioned above supported the analysis of the AD samples. In summary, Oasis 2 is the fastest of the state-of-the-art web applications we could compare to and has no restrictions on the sample number or size.

## Conclusions

Oasis 2 is fast, reliable, and offers several unique features that make it a valuable addition to the ever-growing number of sRNA-seq analysis applications. Especially the analysis support for all organisms, the detection and storage of novel miRNAs, the differential expression and classification modules, and the interactive results visualization supporting GO and pathway enrichment analyses enable biologists and medical researchers to quickly analyze, visualize, and scrutinize their data. Oasis 2 also offers rich per experiment and per sample quality control, which might be one of the most important steps in the initial data analysis. The utility of a good quality control is exemplified in the analysis of the Psoriasis dataset, which seems to contain a mis-labelled (SRR330866_PP) and an outlier (SRR330860_PP) sample (Fig. 3). The removal of the outlier and mis-labelled samples in the Psoriasis dataset increased the number of significantly DE miRNAs from 195 to 256 cases and increased the classification accuracy for the same dataset from AUC of 0.9 to 1. We would like to emphasize that this data was already analyzed in two publications and to our knowledge this is the first time that these ‘problematic’ samples were detected, providing strong evidence for the utility of Oasis 2’ QC plots. Additionally the modular structure of Oasis 2 (sRNA detection, DE and classification) makes this task even easier, as the user can run only DE (without outliers) rather than going through the sRNA detection step again. In addition Oasis 2 provides PDF and video tutorials that explain its usage and details on how to interpret its results. Future developments will include the detection of small RNA editing, modification, and mutation events as well as more detailed reports on bacterial and viral infections and contaminations.

## Additional file

Additional file 1: Oasis2-Suppl-Material.docx: This file contains supplementary material and figures as well. (DOCX 125 kb)
