# Supplementary material for: Oasis 2: improved online analysis of small RNA-seq data
Source: BMC Bioinformatics. 2018 Feb 14;19:54. doi: 10.1186/s12859-018-2047-z (PMC5813365; doi:10.1186/s12859-018-2047-z)
Supplement: Supplementary file 1 — Oasis2-Suppl-Material.docx: This file contains supplementary material and figures as well. (DOCX 125 kb) [file 12859_2018_2047_MOESM1_ESM.docx]

# Supplementary material

1 Implementation Details

## 1.1 **Supported Organisms and Genomes**

Oasis 2 has two modes of analysis in the sRNA detection phase. In case a user selects one of the 14 supported genomes in Oasis 2, reads will be aligned to transcript and genome databases consecutively (Suppl. Table 1 and. Fig. 1). In case the user does not find his organism of interest in the supported genome-list Oasis 2 allows for the analysis of known miRNAs for any organism by aligning reads to all known and novel predicted miRNAs and miRNA families stored in Oasis-DB. Further details on the actual alignment are given in the main text of the manuscript and subsequent supplementary sections.

**Suppl. Table 1.** Oasis 2 supported organisms and genome versions.

| Organism | genome-version | genome-date |
| --- | --- | --- |
| Bos taurus | UMD3.1 | 2009-11 |
| Canis familiaris | CanFam3.1 | 2011-09 |
| Caenorhabditis elegans | WBcel235 | 2012-12 |
| Danio rerio | GRCz10 | 2014-09 |
| Drosophila melanogaster | BDGP6 | 2014-07 |
| Mus musculus | GRCm38 | 2012-01 |
| Gallus gallus | Galgal4 | 2011-11 |
| Gorilla gorilla | gorGor3.1 | 2009-12 |
| Pan troglodytes | CHIMP2.1.4 | 2011-02 |
| Rattus norvegicus | Rnor_6.0 | 2014-07 |
| Homo sapiens | GRCh38 | 2013-12 |
| Sus scrofa | Sscrofa10.2 | 2011-08 |
| Equus caballus | EquCab2 | 2007-09 |
| Anopheles gambiae | agamp4 | 2014-04 |

1.2 Alignment and counting

Before reads are aligned and counted, adapter sequences are trimmed, potential barcode sequences are removed (optional), and reads are filtered for user definable minimum and maximum lengths. In case the data does not contain adapter sequences (e.g. removal prior to upload) the user can skip the adapter removal step. Some recent sRNA library kits add barcodes to reduce the Illumina-generated base bias especially in the first 6 bases of reads (e.g. NEXTflex). Oasis 2 allows for optional barcode removal, in which the user-selected amount of bases is removed from the adapter-free reads on both ends. Subsequent to the adapter trimming and barcode removal, Oasis 2 filters reads for a user-definable minimum and maximum read length (default is 15 and 32, respectively). These minimum and maximum sRNA length settings can be adjusted by the user in Oasis 2’s web interface.

To count the already known target organism-specific (TO) miRNAs in the sample the preprocessed reads are subsequently aligned to all miRNA entries of the specified organism in Oasis-DB (miRBase version 21 and novel predicted miRNAs and miRNA families) using STAR (Dobin et al, 2013) in non-splice-junction-aware mode (Fig. 1, **Step 1**). Reads of length 15 - 19 nucleotides are aligned allowing for 0 mismatches whereas reads of length 20 – 32 nucleotides are mapped allowing for 1 mismatch., a setting that can be adjusted by the user in the sRNA Detection module’s ‘Advanced Options’. In order to count non-miRNA sRNAs the unmapped reads from the previous step are aligned to snRNAs, snoRNAs, rRNAs, and piRNAs in Oasis-DB using the same alignment parameters (Reads of length 15 - 19 nucleotides are aligned allowing for 0 mismatches whereas reads of length 20 – 32 nucleotides are mapped allowing for 1 mismatch) (Fig. 1, **Step 1**).

Unmapped reads from the previous step are aligned to the reference genome to predict novel miRNAs using miRDeep2 (Friedländer et al, 2012) with default parameters (allowing 1 mismatch and no more than five potential genomic target regions) except for the minimum read depth parameter –a (which is automatically computed by Oasis 2 according to the total number of uniquely mapped reads). (Fig. 1, **Step 2**). Novel miRNAs of high quality are automatically added to Oasis-DB and in case of multiple identical genomic regions (not more than 5) a miRNA family is created (see also section 1.4 ‘Oasis-DB miRNA insertion criteria’).

To find potential bacterial, archaeal, or viral sRNA infections or contaminations in samples, all the unmapped reads from **Step 2** are subsequently aligned against a set of 2784 bacterial/archaeal and 4336 viral genomes from the RefSeq database of NCBI (Tatusova et al, 2014) using Kraken (Wood et al, 2014) (Fig. 1, **Step 3**). In more detail, we used a k-mer length of 18 to create a database for viral, bacterial and archaeal genomes using the corresponding script in Kraken. To assign taxonomic labels to an input sequence, Kraken searches all k-mers in the sequence to determine if it exists in any known species. The lowest common ancestor is assigned to sequences which have k-mers in more than one species. Sequences which have no k-mer matches in the database are left as unclassified.

In the last step, all unmapped reads from **Step 3** are aligned without mismatches to all non-target organism miRNAs of Oasis-DB (all of miRBase and all predicted miRNAs) using STAR (Fig. 1, **Step 4**). **Step 4** serves to detect potential orthologous miRNAs.

The exact STAR parameters of the first two alignment steps are as follows:

--outFilterMismatchNoverLmax 0.05 (0 mismatches for reads with length 15-19, 1 mismatch for reads with length 20-32)

--outFilterMatchNmin 15

--outFilterScoreMinOverLread 0

--outFilterMatchNminOverLread 0

--alignIntronMax 1

For the counting step, Oasis 2 assumes un-stranded single end sRNA-seq data and uses the following databases and versions:

- *miRBase* for miRNA sequences (version 21, release date: June 2014)
- piRNAbank V.2 for piRNAs
- Ensembl (version 84) for snRNAs, snoRNAs, and rRNAs.

1.3 Oasis 2 classification module

For the classification, Oasis 2 uses the random forest (RF) binary classification algorithm, implemented in the randomForest R package, as well as cross-validation and feature pruning, implemented in the caret R package. The RF algorithm is known to perform robust in the case of big number of features. However from our experience of its application for sRNA data classification we detected a couple of potential improvements, which we implemented in Oasis 2:

- **Balanced sampling.** In the case of different number of samples in classes the RF cannot perform optimally, making more errors in smaller class samples (actually predicting almost everything as belonging to the bigger class). This can be especially important if the smaller class corresponds to some (rare) disease, on which few data is available.

The way to avoid this issue is to balance numbers of samples in both classes. There are different balancing strategies available (down-sampling, up-sampling, hybrid methods). We decided to use the down-sampling strategy since it is “pessimistic” but avoids “discrimination” of some class and therefore balances misclassification errors between classes.
For that purpose we use stratified sampling from the randomForest R package. It allows specifying the number of samples extracted from each class for construction of random forest trees. We take the size of the smaller class as stratification size.

- **Feature pruning.** The RF selects part of the features for the construction of each tree (mtry parameter, which is by default equal to ⎣√n⎦, where n is total number of features). If there is a big number of non-informative features (“noise”), many trees can be build based on noise only and therefore affect the classification quality.

The way to avoid trees built of noise is feature pruning. There are different pruning strategies available (backwards selection, forwards selection, resampling). The idea is to arrange the variables based on their importance in the full model and then remove less important variables one-by-one, calculating model performance in each step. At the end, the subset of variables with the best performance is considered as optimal. Cross-validation is used for the performance calculation at each step, which gives slightly more reliable results than the OOB error (see ‘Cross-validation error’ list entry).

We use cross-validation-based backwards selection, implemented in the R caret package with 10-fold cross-validation, repeated 10 times at each step for the performance calculation.

- **Cross-validation error.** The RF has an internal performance indicator, the so-called out-of-bag error (OOB), which evaluates the classification performance of the RF on the samples that were not used for construction of its trees. Although the OOB is a decent estimator of the RF error on future samples, it is not always accurate. On the one hand, the OOB may be too optimistic because each sample is excluded from only part of the trees, not from all. On the other hand, the OOB may be too pessimistic since the model may be over-trained on samples used for tree construction and perform badly on the excluded samples used for OOB calculation.

To get more robust performance estimates we now also calculate the cross-validation error next to the OOB error. The idea is to split the available data into training and test sets, train the model on the training set and calculate the accuracy on the test set.

We implemented 10-fold cross validation, repeated 10 times based on randomForest R package and calculate performance measures (error rate, ROC, A.U.C etc.) by averaging errors over that 10 runs.

1.4 Oasis-DB miRNA insertion and naming

- Oasis-DB design: As outlined in the main text of the manuscript and in section ‘1.2 Alignment and counting’ of the supplements, Oasis 2 aligns reads to the target genome to predict novel miRNAs with high-stringency. In order for users to query and retrieve information on these predicted miRNAs, we have created Oasis-DB, a database that stores all relevant information of novel miRNAs. In addition, the database contains all miRBase miRNA entries. In case a miRBase update contains a miRNA with an identical location of a predicted miRNA, Oasis-DB automatically links the predicted miRNA to the corresponding miRBase entry.

Oasis-DB contains the following tables and entries:

**external_mirna** – Information on miRNAs and their precursors from *miRBase*.

**predicted_mirna** – Information on predicted miRNA and their precursors from Oasis 2.

**organism** – Name, genome build, and prefix information for each organism.

**tool** – Version of *miRBase* for external data and software details for predicted miRNAs.

**parameter** – Parameter information of the miRNA prediction software.

**predicted_parameters** – Links predicted miRNAs to the parameters used for its prediction.

**validated_mirna** – Link predicted miRNAs to new *miRBase* entries.

**other_srna** – Information on non-miRNA sRNAs (snRNAs, snoRNAs, rRNAs, and piRNAs).

Oasis-DB is developed using the MySQL open-source relational database management system. The data is made accessible to the users through JAVA web application using java server pages (Jsp) and servlets running on a Tomcat server.

- miRNA insertion and naming : To assure that Oasis-DB contains only high-quality miRNA entries, novel predicted miRNAs have to pass the following criteria:

1. The log-odds score assigned to the hairpin by miRDeep2 (miRDeep2-score) should be greater than 10.
2. The predicted miRNA hairpin should not have sequence similarity to reference tRNAs or rRNAs (rfam alert from miRDeep2 output should be empty).
3. The estimated randfold p-value of the excised potential miRNA hairpin should be equal to or lower than 0.05 (Significant-randfold-p-value from miRDeep2 output should be 'yes').

Novel predicted miRNAs are added to Oasis-DB using the standard nomenclature for miRNAs and the prefix ‘p-’ to indicate predicted. In more detail, following the ‘p-‘ prefix is a 3-4 letter code for the species, 'miR' to represent mature sequences and 'mir' for precursor sequences, followed by a unique numerical identifier for the miRNA (e.g. p-hsa-miR-1). Predicted miRNAs with the same sequence but different genomic locations are considered as miRNA family and obtain a numerical suffix to represent this (e.g. p-mmu-miR-18-1 and p-mmu-miR-18-2 correspond to the same sequence in mouse, but at different locations). It should be noted that orthologous miRNAs and miRNA editing are not yet considered in the naming convention of Oasis-DB.

1.5 Oasis 2 differential expression module

The Oasis 2 ‘DE Analysis’ module features an updated DESeq version and novel interactive visualizations. The core of the analysis algorithms is however very similar to its previous version and the following description is largely congruent with the original publication in Capece *et al.,* 2015.

The ‘DE Analysis’ module accepts count files as input that are output from the ‘sRNA Detection’ module. The output provides various quality metrics, detailed differential expression information, and known and predicted targets of miRNA in an interactive web report. This web report allows for the subsequent functional analysis of the differentially expressed miRNAs by using JavaScript-enabled API queries to GeneMania (interactome and GO analysis) (Zuberi *et al.*, 2013), g:Profiler (GO, pathway-Kegg, Reactome, etc) (Reimand *et al.*, 2011), STRING (protein-protein interaction network) (Franceschini *et al.*, 2013), STITCH (chemical-protein interaction network) (Kuhn *et al.*, 2014), and DAVID (enrichment analysis based on many biological databases) (Huang *et al.*, 2007).

The differential expression analysis provided by DESeq2 (Love *et al.*, 2014) (Supplementary Table 3) supports analyses between multiple groups containing multiple samples. Some of the more advanced comparisons that can be performed using the ‘DE Analysis’ module are:

1. Paired and unpaired analyses (e.g. time-series data)
2. Multiple group comparisons (e.g. control vs. treatment 1 vs. treatment 2)
3. Two or multiple groups including covariates (e.g. control vs. disease including co-variates such as age, gender, and medication).

Whereas the analysis of two or multiple groups is automatically handled by just uploading two or more groups of samples, the covariate analysis requires the upload of covariate information in a separate file. Detailed use cases supplying step-by-step guides for multivariate data analysis are included in the demo datasets.

## 1.6 miRNA target annotation and prediction

Oasis 2 provides validated and predicted miRNA targets for all miRNAs in its database (see also Capece *et al.,* 2015). Known miRNAs are annotated with experimentally validated target RNAs using MirTarBase (Hsu *et al.*, 2014) and miRecords (Xiao *et al.*, 2009) (version 4.5 and version 4 respectively). In addition, known miRNAs are annotated with predicted miRNA targets using the miRanda database (Betel *et al.*, 2008). Oasis 2 supplies only high quality predicted miRNA targets from miRanda by filtering for targets having a ‘good mirSVR score and a conserved miRNA’ or a ‘mirSVR score < -1.2 and a non-conserved miRNA’.

Transcript targets for novel miRNAs are predicted by miRanda 3.3a using the mature miRNA sequence and the complete set of transcript UTRs for a given target organism. In order to minimize the number of false positives present in miRNA target predictions, Oasis 2 uses only conserved sites of transcript UTRs obtained in TargetScan (release 6.2) alignment (Friedman *et al.*, 2009; Witkos *et al.*, 2011). Only targets with alignment scores higher than 155 and energy scores lower than -20 are considered. The list of predicted targets is sorted and displayed in decreasing score order.

1.7 Software and tools used in Oasis 2

The following tables list the software and version information that are used in the different analysis modules of Oasis 2.

**Supplementary Table 2.** Software used by the ‘sRNA Detection’ module.

| Task | Software/Tool | Version |
| --- | --- | --- |
| Adapter trimming | Cutadapt (Martin, 2011) | 1.7.1 |
| Alignment | STAR (Dobin et al, 2013) | 2.4.1d |
| miRNA prediction | mirDeep2 (Friedländer et al, 2012) | 2.0.0.5 |
| Quality assessment | FastQC (Andrews) | 0.11.2 |
| Counting | featureCounts (Liao et al, 2014) | 1.4.6 |
| Pathogen detection | Kraken (Wood et al, 2014) | 0.10.5-beta |

**Supplementary Table 3.** Software used by the ‘DE Analysis’ module.

| **Task** | **Software** | **Version** |
| --- | --- | --- |
| **Differential expression** | **DESeq2 (Love et al., 2014)** | **1.16** |
| **Target prediction/annotation** | **miRanda (Betel et al., 2008)**  MirTarBase (Hsu *et al.*, 2014)  miRecords (Xiao *et al.*, 2009) | **3.3a**  **4.5**  **4.0** |
| **Functional analysis** | **GeneMania (Zuberi et al., 2013)**  **G:Profiler (Reimand et al., 2011)**  **STRING (Franceschini et al., 2013)**  **STITCH (Kuhn et al., 2014)**  **DAVID (Huang et al., 2007)** | **-**  **-**  **-**  **-**  **-** |
|  |  |  |
|  |  |  |
|  |  |  |
|  |  |  |

**Supplementary Table 4.** Software used by the ‘Classification’ module.

| **Task** | **Software/Package** | **Version** |
| --- | --- | --- |
| **Normalization** | DESeq2 (Love et al, 2014) | 1.4.5 |
| **Classification** | randomForest (Liaw & Matthew, 2002) | 4.6-10 |
| **Visualization** | ROCR (Sing et al, 2005) | 1.0-5 |

**Supplementary Table 5.** Software used by the ‘Search’ module.

| **Task** | **Software/Package** | **Version** |
| --- | --- | --- |
| **Data storage** | MySQL | 5.1.73 |
| **Data Search** | JAVA/EE | 1.7 |

1.8 Batch job submission (API)

Oasis 2 API is written in Python and it has been tested with Python 3. It contains 3 main scripts: ***oasisSRNA***.py (mapping, predict novel miRNA and quality control), ***oasisDE***.py (differential expression analysis) and ***oasisClassification***.py (classification analysis). They can be downloaded in the main webpage of Oasis 2.

These scripts send the data to the server for one of the three specific analyses. On the server-side there are three small web services to accept connections, one for each type of analysis. These web services only accept HTTP post method in multipart/form-data.

***oasisSRNA.py***

This script sends compressed sRNA FASTQ files to the server and supplies the parameters needed for the ‘sRNA Detection’ analysis. In brief, after checking for the command line arguments, the script assures that the web-application is available and in case of communication problems reports an error and exits. Subsequently the files are tested for compliance and an HTTP post request sending a multipart-encoded file is dispatched. In case the user submits several samples with the same settings the process is repeated for every file.

***oasisDE.py***

This script sends count files to the server and supplies the parameters needed for the ‘DE Analysis’ module. Analogous to ‘***oasisSRNA***.py’, the script tests for a valid connection and file validity and sends an HTTP post request to the server. The script supports basic two group analyses as well as multivariate analyses, supporting the full functionality of the ‘DE Analysis’ module.

***oasisClassification.py***

This script sends count files to the server and supplies the parameters needed for the ‘Classification’ module. Analogous to ‘***oasisDE***.py’, the script tests for a valid connection and file validity and sends an HTTP post request to the server.

2 Additional figures and tables

**Predicted**

**miRNAs**

**(769)**

**Suppl. Fig. 1.** Predicted miRNAs in Oasis 2 as of November 2017. Predictions are based on the miRDeep2 software and require a score of at least 10 to be considered as potential high-quality miRNA predictions.


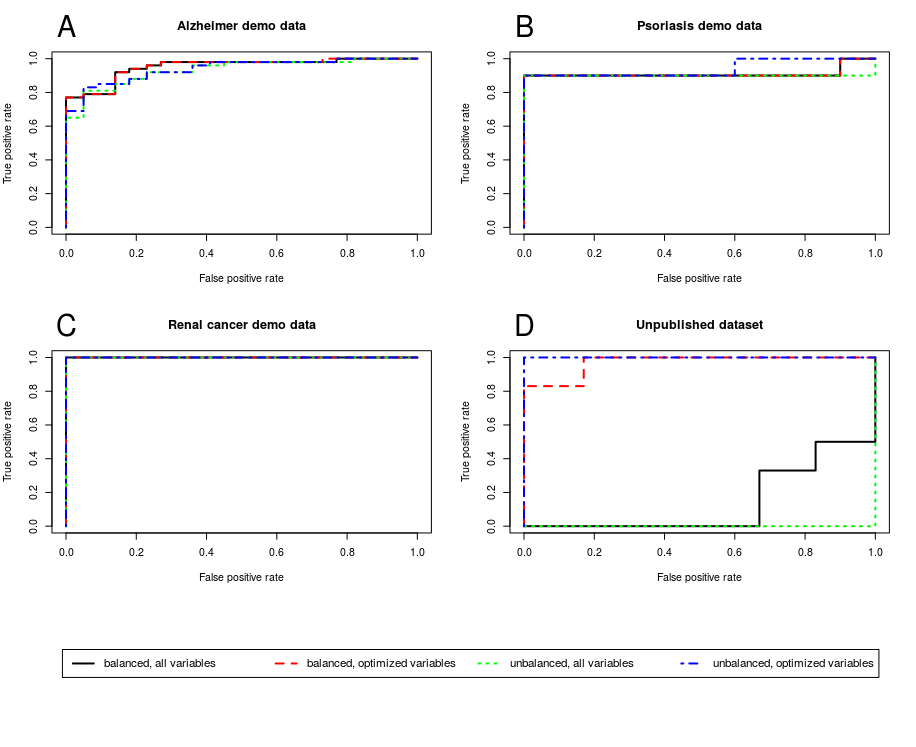


**Suppl. Fig. 2.** Prediction performance on the demo datasets. The prediction is shown for balanced/unbalanced sampling as well as for all variables/optimized set of variables. The old Oasis classification module performed unbalanced sampling with all variables (green dotted line). The combination of balancing and optimization of number of variables (blue dash-dotted line) in the novel RF classification module improves the classification performance. The novel classification module is robust to class imbalances and effectively filters for informative features while removing non-informative ones. For the demo datasets (A,B,C) the improvement is consistent albeit not big (2% for A, 1% for B, no improvement for C), however for the unpublished sRNA-seq disease dataset (D) it improves from full misclassification to completely correct classification.

**Supplementary Table 6.** Datasets with defined viral or bacterial infections

| GSEID | Title |
| --- | --- |
| GSE59944 | In depth analysis of the interaction of HIV-1 with cellular microRNA biogenesis and effector mechanisms (Whisnant et al, 2013) |
| GSE65752 | Modulation of the microRNA cluster miR-183-96-182 expression by the Epstein-Barr virus latent membrane protein 1 (Oussaief et al, 2015) |
| GSE31349 | microRNA profiling in Marek's disease virus induced lymphoma and infected spleen by deep sequencing (Lian et al, 2013) |
| GSE33584 | High-resolution profiling and analysis of viral and host small RNAs during human cytomegalovirus infection (Stark et al, 2012) |
| GSE72769 | High-throughput small RNA-sequencing of human macrophages infected with Mycobacterium abscessus Smooth and Rough variants (Dinan et al, 2015) |

**Supplementary Table 7.** Runtime of different Oasis 2 modules.

| **Demo Dataset** | **Oasis 2 Compression** | **Oasis 2**  **sRNA Detection^1^** | **Oasis 2**  **DE Analysis** | **Oasis 2 Classification** |
| --- | --- | --- | --- | --- |
| **AD (287 GB)** | 4h32m | 3h46m | 5m29s | 8m21s |
| **Psoriasis (48 GB)** | 37m12s | 52m | 3m17s | 2m48s |
| **Renal Cancer (9 GB)** | 12m01s | 15m | 2m18s | 2m24s |

^1^ Runtime estimates include the decompression of the data on the server side.

## Supplementary references

Andrews. (n.d.). *FastQC A Quality Control tool for High Throughput Sequence Data*. Retrieved from http://www.bioinformatics.babraham.ac.uk/projects/fastqc/

Betel et al. (2008). The microRNA.org resource: Targets and expression. *Nucleic Acids Res*.

Capece et al. (2015). Oasis: online analysis of small RNA deep sequencing data. *Bioinformatics*.

Dinan et al. (2015). High-throughput small RNA-sequencing of human macrophages infected with Mycobacterium abscessus Smooth and Rough variants.

Dobin et al. (2013). STAR: ultrafast universal RNA-seq aligner. *Bioinformatics*, 15-21.

Franceschini et al . (2013). STRING v9.1: protein-protein interaction networks, with increased coverage and integration. *Nucleic Acids Res*, 41 , D808–D815.

Friedländer et al. (2012). MiRDeep2 accurately identifies known and hundreds of novel microRNA genes in seven animal clades. *Nucleic Acids Res*, 37-52.

Huang et al. (2007). The DAVID Gene Functional Classification Tool: a novel biological module-centric algorithm to functionally analyze large gene lists. *8*, R183.

Joyce et al. (2011). Deep sequencing of small RNAs from human skin reveals major alterations in the psoriasis miRNAome. *Hum. Mol. Genet, 20*, 4025–40.

Kuhn et al . (2014). STITCH 4: Integration of protein-chemical interactions with user data. *Nucleic Acids Res*, 42.

Leidinger et al. (2013). A blood based 12-miRNA signature of Alzheimer disease patients. *Genome Biol, 14*, R78.

Lian et al. (2013). microRNA profiling in Marek's disease virus induced lymphoma and infected spleen by deep sequencing.

Liao et al. (2014). featureCounts: an efficient general purpose program for assigning sequence reads to genomic features. *Bioinformatics*.

Liaw & Matthew. (2002). Classification and Regression by randomForest. *R News, 2*, 18–22.

Love et al. (2014). Moderated estimation of fold change and dispersion for RNA-Seq data with DESeq2. *Genome Biology*.

Love et al. (2014). Moderated estimation of fold change and dispersion for RNA-Seq data with DESeq2. *bioRxiv*.

Martin. (2011). Cutadapt removes adapter sequences from high-throughput sequencing reads. *EMBnet.journal*.

Osanto et al. (2012). Genome-wide microRNA expression analysis of clear cell renal cell carcinoma by next generation deep sequencing. *PLoS One, 7*.

Oussaief et al. (2015). Modulation of MicroRNA Cluster miR-183-96-182 Expression by Epstein-Barr Virus Latent Membrane Protein 1. *J Virol*, 12178-88.

Reimand et al. (2011). G:Profiler - A web server for functional interpretation of gene lists (2011 update). . *Nucleic Acids Res*.

Robinson et al. (2010). edgeR: a Bioconductor package for differential expression analysis of digital gene expression data. *Bioinformatics, 26*, 139–40.

Sing et al. (2005). ROCR: Visualizing classifier performance in R. *Bioinformatics, 21*, 3940–3941.

Stark et al. (2012). High-resolution profiling and analysis of viral and host small RNAs during human cytomegalovirus infection. *J Virol, 86(1)*, 226-35.

Tatusova et al. (2014). RefSeq microbial genomes database: new representation and annotation strategy. *Nucleic Acids Research, 42*, D553–D559.

Whisnant et al. (2013). In-depth analysis of the interaction of HIV-1 with cellular microRNA biogenesis and effector mechanisms. *MBio*.

Wood et al. (2014). Kraken: ultrafast metagenomic sequence classification using exact alignments. *Genome Biology, 15(3)*, R46.

Zuberi et al. (2013). GeneMANIA prediction server 2013 update. *Nucleic Acids Res*.
